# Joint genetic analysis of hippocampal size in mouse and human identifies a novel gene linked to neurodegenerative disease

**DOI:** 10.1186/1471-2164-15-850

**Published:** 2014-10-03

**Authors:** David G Ashbrook, Robert W Williams, Lu Lu, Jason L Stein, Derrek P Hibar, Thomas E Nichols, Sarah E Medland, Paul M Thompson, Reinmar Hager

**Affiliations:** Computational and Evolutionary Biology, Faculty of Life Sciences, University of Manchester, Michael Smith Building, Oxford Road, Manchester, M13 9PT UK; University of Tennessee Health Science Center, Memphis, TN 38163 USA; Jiangsu Key Laboratory of Neuroregeneration, Nantong University, Nantong, China; Department of Neurology, Laboratory of Neuro Imaging, UCLA School of Medicine, Los Angeles, CA 90095-1769 USA; Institute for Neuroimaging and Informatics, Imaging Genetics Center, Keck School of Medicine, University of Southern California, 2001 N. Soto Street, Los Angeles, CA 90033 USA; Department of Statistics & Warwick Manufacturing Group, The University of Warwick, Coventry, CV4 7AL UK; Genetic Epidemiology Laboratory, Queensland Institute of Medical Research Berghofer, Brisbane, Australia

**Keywords:** Comparative analysis, Hippocampus, MGST3, BXD

## Abstract

**Background:**

Variation in hippocampal volume has been linked to significant differences in memory, behavior, and cognition among individuals. To identify genetic variants underlying such differences and associated disease phenotypes, multinational consortia such as ENIGMA have used large magnetic resonance imaging (MRI) data sets in human GWAS studies. In addition, mapping studies in mouse model systems have identified genetic variants for brain structure variation with great power. A key challenge is to understand how genetically based differences in brain structure lead to the propensity to develop specific neurological disorders.

**Results:**

We combine the largest human GWAS of brain structure with the largest mammalian model system, the BXD recombinant inbred mouse population, to identify novel genetic targets influencing brain structure variation that are linked to increased risk for neurological disorders. We first use a novel cross-species, comparative analysis using mouse and human genetic data to identify a candidate gene, *MGST3,* associated with adult hippocampus size in both systems. We then establish the coregulation and function of this gene in a comprehensive systems-analysis.

**Conclusions:**

We find that *MGST3* is associated with hippocampus size and is linked to a group of neurodegenerative disorders, such as Alzheimer’s.

**Electronic supplementary material:**

The online version of this article (doi:10.1186/1471-2164-15-850) contains supplementary material, which is available to authorized users.

## Background

The hippocampus is a key forebrain region involved in declarative memory, cognition, and spatial navigation. Hippocampal volume is highly variable with unilateral values ranging from ~2500 to 5000 mm^3^ among healthy young humans (mean 3,917 mm^3^, s.d. = 441 mm^3^) and from 15.2 to 23.0 mm^3^ among young adult mice [1, 2]. Heritability ranges from 40% to 70% in both species [3, 4], and a small fraction of the difference in volume is also attributable to sex [4, 5]. This wide range of natural variation raises the possibility that susceptibility to a subset of neurodegenerative and psychiatric disorders linked to defects in the hippocampus may depend, in part, on its initial healthy volume. Individuals who develop and retain a large hippocampus into adulthood may be comparatively resistant to some forms of disease, particularly Alzheimer’s. Such a "reserve" hypothesis of neurological disease [6, 7] has been proposed for Parkinson’s [8], Huntington’s [9] and Alzheimer’s [10] diseases. Lower than average volume has been linked to a number of disorders [11] including depression [12–16], Alzheimer’s disease [17] and schizophrenia [18]. Understanding the genetic factors that contribute to individual differences in hippocampal volume is thus crucial in providing insight into vulnerability and severity of disease.

Prior efforts to identify genetic variants underlying differences in brain structure have used large data sets in human genome-wide association studies (GWAS) or extensive mapping populations in mouse model systems. GWAS in humans typically have modest statistical power due to high corrections needed to compensate for multiple testing. However, loci are defined with very high precision, often down to the level of single nucleotide polymorphisms (SNPs). In contrast, mouse linkage studies often have high statistical power to detect genetic effects but lower genetic resolution, producing loci that include hundreds of genes [19, 20]. Combining data from mice and humans overcomes some of these problems, gaining power from mouse crosses and precision from human GWAS. This method also ensures the translational relevance, giving confidence to the human results, as the same gene controlling the same phenotype is found in a related species. Further, this approach illustrates that the homologous mouse gene is relevant to the human phenotype, as well as the significance of experimental research in model systems that would not be possible in humans. Homologous genes are genes that share a common evolutionary ancestor. In this study we are specifically looking at a subset of homologous genes, orthologs, which derive from a speciation event, rather than paralogs, which arise because of a gene duplication event.

This study takes a cross-species approach to identify genes with an evolutionarily conserved role in influencing hippocampus size; i.e. because a given gene is playing the same role in two different species we hypothesize that it was playing the same role in the ancestral species. Previous studies have begun to show the utility of using a cross-species approach to identify genes underlying a phenotype of interest [21–25]. This approach has the advantage that it allows the investigation of disease phenotypes without requiring data from experimental perturbations. Instead we utilize data obtained from populations that segregated for large numbers of common sequence variants and associated differences in phenotype.

Here, we use data from the largest mouse model system, BXD, to identify a set of genes associated with hippocampus size in a joint analysis with human hippocampus MRI data obtained by the ENIGMA consortium for GWAS [26]. We identify, *MGST3* [Entrez: 4259] and use a systems-genetics approach that links this gene to neurodegenerative disorders such as Alzheimer’s disease and Parkinson’s disease.

## Results

### Identification of genes significant in both species

Associations between genes and hippocampus size in BXD mice were estimated using p-values for over 3800 markers obtained for QTL interval mapping. QTL mapping identifies a region of the genome significantly linked to variation in the phenotype. Having identified QTL, we then estimated a particular gene’s significance based on its base pair distance from the nearest two markers and the significance of these two markers. Therefore any particular gene will have a p-value somewhere between the p-values of its two closest markers. The next step in our analysis was to obtain SNP level p-values for association with human hippocampus volume, which were converted to gene p-values to allow comparison with data for the mouse hippocampus. Using the SNP p-values from the human GWAS, the Versatile Gene-based Association Study (VEGAS) website [27] produced gene p-values for a total of 17,787 human genes. Secondly, the mouse homologues of these human genes were identified and yielded a total of 15,705 genes (88.3% of the human genes).

**Figure 1:**
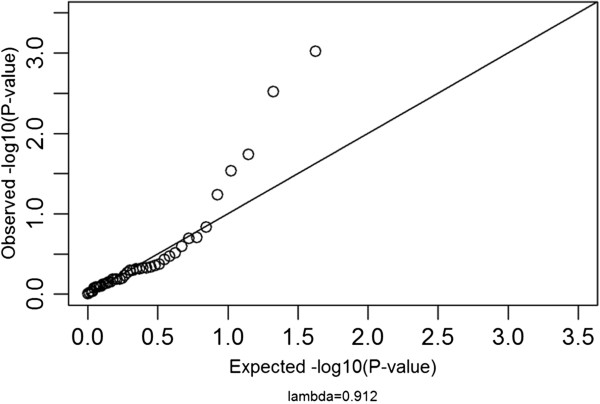
**Quantile-quantile plot of human homologues of significant mouse genes for hippocampus size.** For genes with a significant influence on hippocampus weight in mice (≤0.05) the significance of their influence on human hippocampus volume was plotted against a normal distribution of p-values. Although there are outliers, most of the points lie close to the y = x line, indicating there is no difference between what is seen in the data and what would be expected by chance. This is reinforced by the non-significant lambda value close to 1, which indicates no inflation of significance values.

Using a relaxed (i.e. uncorrected) p-value of ≤ 0.05, 1015 human genes (916 with mouse homologues; 90.2%) were then nominally identified as having an effect on hippocampus size. Overall, there is no indication that the significance of any given gene with the entire region identified in the QTL analysis of BXD mouse hippocampus weight is indicative of the homologous gene’s significance on human hippocampus volume, as judged by the quantile-quantile plot and lambda (λ = 0.912, p = 0.82; Figure 1). This is corroborated by a separate Rank Rank Hypergeometric Overlap test [28] used to compare the two datasets, which yielded a non-significant result (p = 0.38, corrected by the familywise error rate). This is unsurprising as a QTL analysis identifies a region of the genome associated with a trait, and therefore in our analysis all genes within the mouse QTL were significant. However, not all the genes within a QTL contribute to the phenotype, but only a subset or even a single gene.

Therefore we sought to identify which genes are associated with both BXD mouse hippocampus weight and human hippocampus volume. The 42 genes which were significant in mouse are all within a QTL on chromosome 1 [4, 29].Only one gene had a significant human p-value when corrected for multiple testing (0.05/42 = p ≤ 0.0012; Additional file 1), *MGST3*.

### Regulation of gene expression

To establish if our candidate gene was regulating its own expression, mouse hippocampus microarray data from GeneNetwork was used to find probes corresponding to expression of the gene, and WebQTL was used to produce eQTL [30]. Of the 17 probes for *Mgst3* [Entrez: 66447] within the exon array data, six have a significant Pearson’s correlation (r ≥ 0.5, p ≤ 0.05), and these probes were used to represent expression of *Mgst3* in the mouse hippocampus. The six probes represent all four probes for exons and one each from the 5′ and 3′ UTR (Table 1). The remaining 11 probes were for introns and UTRs. This shows that the correlating probes represent the protein coding parts of the gene.

**Table 1:** **Pearson correlations between probes for** ***Mgst3*** **in adult mouse hippocampus**

	Probe ID	Location (Mbp)	Target	1	2	3	4	5	6
1	4654447	169.302579	3′UTR	1	r = 0.921	r = 0.873	r = 0.787	r = 0.8	r = 0.62
					p < 1E-16	p < 1E-16	p < 1E-16	p < 1E-16	p = 9.87E-10
2	5358488	169.302648	exon5	r = 0.921	1	r = 0.903	r = 0.84	r = 0.828	r = 0.632
				p < 1E-16		p < 1E-16	p < 1E-16	p < 1E-16	p = 3.71E-10
3	5399827	169.303925	exon4	r = 0.873	r = 0.903	1	r = 0.937	r = 0.884	r = 0.534
				p < 1E-16	p < 1E-16		p < 1E-16	p < 1E-16	p = 5.33E-7
4	5566068	169.307412	exon2	r = 0.787	r = 0.84	r = 0.937	1	r = 0.92	r = 0.512
				p < 1E-16	p < 1E-16	p < 1E-16		p < 1E-16	p = 1.84E-6
5	5025657	169.308444	exon1	r = 0.8	r = 0.828	r = 0.884	r = 0.92	1	r = 0.519
				p < 1E-16	p < 1E-16	p < 1E-16	p < 1E-16		p = 1.24E-6
6	5280988	169.323882	5′UTR	r = 0.62	r = 0.632	r = 0.534	r = 0.512	r = 0.519	1
				p = 9.87E-10	p = 3.71E-10	p = 5.33E-7	p = 1.84E-6	p = 1.24E-6	

Pearson product-moment correlations produced by GeneNetwork. The dataset used was UMUTAffy Hippocampus Exon (Feb09) RMA (GN206). All probes are for *Mgst3* located on chromosome 1.

*Mgst3* has a *cis*-eQTL, suggesting it regulates its own expression. No *trans*-QTL was found which was consistent between probes. The QTL and eQTL analysis also showed that the C57BL/6 J (B6) allele increased hippocampus weight, whereas the DBA/2 J (D2) allele increased the expression of *Mgst3*.

### Functional analysis of significant genes

To investigate the function of our candidate genes, we used the Database for Annotation, Visualization and Integrated Discovery (DAVID) as it allows us to analyse a number of different annotation databases. Significance was determined by the false discovery rate (FDR), which corrects the significance value for the large number of multiple comparisons.

To determine if any annotations were enriched in both mouse and human, even though individual genes were not shared, separate lists of genes nominally significant in human (915 genes p ≤ 0.05) and human homologues of the 42 mouse genes with p ≤ 0.05 were entered into DAVID. No overlapping significant annotations were found, i.e. no annotations were significantly enriched in both the genes significant in human and the genes significant in mouse. Again, this shows that not all 42 genes within the mouse QTL influence the phenotype, but only a subset.

### Guilt-by-association

Coexpression of genes implies that they share the same regulatory mechanisms [31] and/or are involved in the same biological processes. A ‘guilt-by-association’ approach asserts that the function of a gene, or list of genes, can be indicated by the genes that it commonly coexpresses with, as common coexpression indicates they are part of the same biological process [32]. The large datasets of gene expression provided by GeneNetwork and GeneFriends allows this ‘guilt-by-association’ approach to be used. This is especially useful for genes such as *MGST3/Mgst3*, which previously have not been investigated in detail.

GeneFriends shows human genes which coexpress in a large number of datasets from the Gene Expression omnibus. However it is not specific for tissue or treatment. This identified 8135 genes that were coexpressed with *MGST3* in over half of the datasets (coexpression value ≥ 0.5; Additional file 2). These were analysed using DAVID, to find what KEGG pathway annotations were significant (FDR ≤ 0.05; Additional file 3). One of the six KEGG pathways is particularly interesting; Alzheimer’s disease (FDR = 0.0029).

To support a specific link between genes that are coexpressed with *Mgst3* and Alzheimer’s disease we used the exon array data from GeneNetwork, as this is specific to the hippocampus. Each of the six above identified probes for *Mgst3* was correlated against the entire exon array dataset (1,236,087 probes) to find the top 20,000 probes with which it correlates. These six lists of probes were then combined to find which probes correlated with all six probes for *Mgst3*. This produced a list of 5906 probes which correlated with all six of the probes for *Mgst3*, representing 2971 genes (Additional file 4). Permutation testing was carried out to determine an empirical p-value for how often six lists of 20,000 values from a choice of 1,236,087 values would overlap, and produced a p-value of < 1 × 10^-6^. This shows that the overlap between our probes is highly significant, and that these 2971 genes really do coexpress with *Mgst3* in the mouse hippocampus. Enrichment of this list of genes was then examined in DAVID, and again we see genes involved in neurodegenerative diseases significantly enriched (Additional file 5): Huntington’s disease (95 genes, FDR = 3.29E-27, Parkinson’s disease (77 genes, FDR = 1.56E-25) and Alzheimer’s disease (83 genes, FDR = 1.29E-18).

Finally, the overlap between the genes that are commonly coexpressed with *MGST3* and human homologues of the genes that are coexpressed with *Mgst3* in the mouse hippocampus was examined. This showed that 1579 genes which commonly coexpress with *MGST3* also coexpress with its mouse homologue in the mouse hippocampus (Additional file 6). We tested this by permutation taking samples of 8135 genes and 2971 genes from a list of all known human protein coding genes and determining how often an overlap larger than 1579 was seen. This produced an empirical p-value ≤ 1 × 10^-6^. Again with KEGG enrichment analysis, the three neurodegenerative diseases are highly significant (Additional file 7): Huntington’s disease (78 genes, FDR = 3.08E-22), Parkinson’s disease (63 genes, FDR = 5.68E-21) and Alzheimer’s disease (69 genes (FDR = 1.34E-18).

## Discussion

We found strong evidence that *MGST3,* on chromosome 1 in both mouse and human, is significantly associated with hippocampus size. MGST3 has previously been found to be down-regulated in Alzheimer’s disease [33]. The fact that the gene appears to have an evolutionarily conserved role in both species suggests a role in hippocampus morphology. MGST3 has been found to be particularly highly expressed in the rat hippocampus [34]. A ‘guilt-by-association’ approach shows that these genes coexpress with genes linked to neurodegenerative disorders associated with reduced hippocampus volume: Huntington’s disease [35–37], Alzheimer’s disease [17, 38] and Parkinson’s disease [39–41].

The potential mechanism for this link is more speculative. Genes that coexpress with *MGST3* are also associated with cellular energy production, as the oxidative phosphorylation KEGG pathway appears in our results [Additional files 3, 5 and 7]. Mitochondrial dysfunction has been implicated in both neuropsychiatric and neurodegenerative disorders [42, 43], linking the mitochondrial and neurodegenerative annotations. Recently it has been reported that dysfunction of mtDNA genes, which have been implicated in Alzheimer’s disease, directly influence left hippocampal atrophy [44]. Further, links have also been found between oxidative stress and regulation of *Mgst3* in mice [45].

MGST3 has also been linked to inflammation, as it and other family members show leukotriene C4 (LTC_4_) synthase activity. Leukotrienes are physiological important mediators of various inflammatory and immediate hypersensitivity processes [46]. When porcine kidney cells were exposed a nephrotoxin, aristolochic acid I, MGST3 and FLAP (another family member) were upregulated before an increase in LTs synthesis. This is relevant as Alzheimer’s disease, as well as other neurodegenerative disorders, have been linked to inflammation (reviewed in [47]). However, other research has found that rat MGST3 does not have LTC_4_ synthase activity [48], is not upregulated in response to lipopolysaccharide [49] and does not appear to be directly involved in the inflammation response [34]. In this last paper, the authors speculate that it may have a neuroprotective role against oxidative stress [34].

## Conclusions

In summary, the combination of human GWAS and mouse QTL data from some of the largest study systems available has enabled us to identify a novel gene, *MGST3,* which is associated with hippocampus size across species and, when dysregulated, is linked to neurodegenerative disorders.

## Methods

### Data

We used mouse hippocampus weight data from 35 BXD lines plus the parental C57BL/6 J and DBA/2 J strains, adjusted for age, sex, body weight, and brain weight minus hippocampus weight [4, 30] (GN13031). Over 3800 SNP markers are used for QTL interval mapping, thus for each marker significance values are available. Using the gene’s base pair distance from the nearest two markers we developed a python script [50] to produce an estimated p-value for each gene. For example a gene positioned halfway between two markers would have an estimated p-value half way between the two marker values. Therefore an estimated p-value could be produced for any gene in the mouse genome (NCBI37/mm9) by using the gene’s known start position and any set of mouse markers.

Human MRI-generated hippocampus volume from healthy subjects and patients was generated for GWAS meta-analyses by the Enhancing Neuro Imaging Genetics Through Meta-Analysis (ENIGMA) network [1, 26, 51] and can be visualised with ENIGMAvis [51]. Association analyses used multiple linear regression with hippocampus volume as a dependent variable and the additive dosage of each SNP as an independent variable, controlling for covariates of population stratification (four MDS components), intracranial volume, age, age^2^, sex and the interactions between age and sex and age^2^ and sex. Dummy covariates were used to control for different scanner sequences or equipment within a site. We converted the p-values available for each SNP marker to gene level significance values using the Versatile Gene-based Association Study [27] website (VEGAS). This tool tests for association between the phenotype and a gene by summarizing the full set of SNPs in the gene. Linkage disequilibrium between SNPs in a gene is taken into account by using simulation based on the pre-calculated linkage disequilibrium structure of a set of reference individuals from the HapMap phase 2 CEU population. SNPs are assigned to each of 17,787 autosomal genes on the UCSC Genome Browser hg18 assembly, with boundaries defined as ±50 kb of 5′ and 3′ UTRs. Association p-values for any given gene with n SNPs are converted to uppertail chi-squared statistics with one degree of freedom (df). The gene-based test statistic is then the sum of all of the chi-squared 1 df statistics within that gene. If the SNPs are in perfect linkage equilibrium, the test statistic will have a chi-squared distribution with n degrees of freedom under the null hypothesis. However this is unlikely to be the case, therefore the true null distribution given the LD structure (and hence p-values that correlate accordingly) will need to be taken into account. This is done by simulating a large number of multivariate normal vectors, and the empirical gene based p-value is the proportion of simulated test statistics that exceed the observed gene-based test statistic [27]. Thus, we are able to identify genes associated with hippocampus size that may be significant, independent of whether individual SNPs are significant.

All data used for the above is from existing, previously published, anonymised data and therefore no further ethical approval was needed.

### Identification of significant genes for hippocampus size in mouse and human

To be able to compare the data between species, mouse homologues for the human genes need to be identified. The marker method above can produce a p-value for any mouse gene, therefore it is the human genes produced by VEGAS that limit the total number of genes in our analysis. Using the human SNP p-values, VEGAS produced p-values for 17,787 human genes (the number of autosomal genes on the UCSC Genome Browser hg18 assembly). We used several tools to identify mouse homologues for these human genes: MammalHom [52], Mouse Genome Informatics [53] and HomoloGene [54]. Thus, 15,705 mouse genes with a corresponding human homologue were identified, representing 88.3% of human genes.

To determine if genes affecting hippocampus weight in mouse also influence hippocampus volume in humans we used a protocol developed in R [55]. Firstly, we produced a quantile-quantile plot using the human p-values of those of the 42 genes which had a mouse p-value of ≤ 0.05. Secondly, the genomic control λ-value [56] was calculated. This value is generally a measure of inflation of statistics due to population stratification, i.e. if significance is increased due to the populations being related. In our case a high lambda would show that overall those genes with a significant mouse p-value have a higher human p-value than would be expected by chance. In other words, by using genes which are significant in mouse, the p-values of the homologous human genes would be inflated. In our study we tested this value by permutations, with the same number of random genes sampled from the genome and the λ-value calculated (random λ). The number of times that the random λ was greater than the calculated λ was divided by the number of permutations (100,000) to give the p-value of the calculated lambda values. The permutations determine if a high λ-value is simply due to an overall high λ between the two datasets, i.e. that all the p-values in human are higher than would be expected by chance. We validated results thus obtained using an additional approach, the Rank Rank Hypergeometric Overlap test [28]. This was carried out using the RRHO R package [57], which computes the number of overlapping elements, and return the observed significance of this overlap using a hypergeometric test.

Thirdly, to assess if any particular gene is associated with brain region size in both mouse and human the significance of the homologues for the 42 genes found to be significant in BXD mice were examined in the human GWAS data. This was corrected for multiple comparisons using the number of genes compared [23] (42 significant mouse genes), therefore 0.05/42 = p < 0.0012.

### Expression quantitative trait loci

Expression quantitative trait loci (eQTL) show regions of the genome that influence the expression of a gene of interest. A *cis*-eQTL, i.e. an eQTL in the same position of the candidate gene, suggests that the candidate gene regulates its own expression, whereas a *trans*-eQTL, i.e. a QTL elsewhere in the genome, indicates that a gene at this position is influencing the expression of the candidate gene. Data for exon mRNA expression in the hippocampus of mouse lines (mainly BXD but with data from other inbred mouse lines) available at GeneNetwork were used and WebQTL [58] produced eQTL for genes identified above [30]. The database of microarray results used from GeneNetwork was UMUTAffy Hippocampus Exon (Feb09) RMA (GN206) [59]. This allows the examination of the *cis*- or *trans*- regulation of our identified gene (*Mgst3*) in the mouse hippocampus. Using this exon gene expression data, all probes for *Mgst3* were correlated using Pearson’s product-moment correlation as implemented in GeneNetwork, and those probes which showed a significant correlation (r ≥ 0.5, p ≤ 0.05) were said to represent the expression of the gene.

### Functional analysis

Functional analysis allows us to investigate enrichment; for example if the molecular function of a gene product is over-represented in a submitted list of genes. Enrichment therefore suggests whether a particular gene or a set of genes is associated with a particular function or disorder.

The Database for Annotation, Visualization and Integrated Discovery (DAVID) [60, 61] identifies if a given list of genes is significantly enriched in an annotated gene term. DAVID uses a range of databases, including Gene Ontologies (GO) terms [62], Kyoto Encyclopedia of Genes and Genomes (KEGG) pathways [63], Online Mendelian Inheritance in Man diseases and InterPro protein domains [64]. Separate lists of all significant mouse genes (p ≤ 0.05) and all nominally significant human genes (p ≤ 0.05) were analysed, and the results examined for any annotations that appeared in both datasets. The latter would suggest that the same pathways or networks were involved in the phenotype in both species, even if the same individual genes are not significant.

### Coexpresion and ‘Guilt-by-association’

Shared regulation and function of genes can also be established using coexpression analysis [31]. However, coexpression can differ between species or between tissues within an organism. To examine if genes are commonly coexpressed in humans, GeneFriends can be used [32]. GeneFriends takes submitted list of genes and uses a large database of microarray data (4164 Micro array datasets containing 26,113 experimental conditions and 19,080 genes) [32], from the Gene Expression Omnibus [65, 66] to find genes that are commonly coexpressed with the entered gene list. However it is not specific for tissue type or treatment, and therefore can only inform us which genes tend to coexpress together, and not which genes specifically coexpress in the hippocampus or at what time points.

GeneFriends produces a list of genes that are coexpressed with the submitted genes in a significant number of datasets, to identify commonly coexpressed genes (coexpressed independent of treatment or tissue). Common coexpression suggests that the genes are under the same regulation in particular since coexpression is analysed across conditions and tissues. This list of commonly coexpressed genes was analysed using DAVID as above, producing annotations for these genes. This allows a ‘guilt-by-association’ approach, where the roles played by genes that are commonly coexpressed with our genes are used to suggest the networks that the genes are part of [32]. We next used Pearson product-moment correlations, as implemented in GeneNetwork, to examine coexpression in mice by producing correlation matrices of hippocampal gene expression [67]. In contrast to GeneFriends this is specific to the hippocampus. Hippocampus mRNA expression was found for *Mgst3* in the UMUTAffy Hippocampus Exon (Feb09) RMA (GN206) microarray database. The probes for *Mgst3* were correlated with each other, and six showed a significant correlation (r ≥ 0.5, p ≤ 0.05) and were used to determine gene expression. For each of these six correlating probes for *Mgst3*, the top 20,000 correlations were then found within the whole hippocampus exon array dataset (1,236,087 probes). 5906 probes correlated with all six of the probes for *Mgst3,* representing 2971 genes. This list of 2971 was submitted to DAVID to determine KEGG pathway enrichment. Significance testing using permutations was then carried out to determine the overlap between six random samples of 20,000 values (the number of correlations) from a total of 1,236,087 values (the total number of probes). With 1,000,000 permutations this produced a value of p < 1 × 10^-6^.

The overlap between the genes identified by GeneFriends and those identified by GeneNetwork was also examined. The resulting list of genes was then submitted to DAVID for KEGG pathway enrichment analysis. The significance of the number of overlapping genes was again determined by permutation. Samples of sizes 8135 (the number of coexpressing genes found by GeneFriends) and of 2971 (the number of coexpressing genes found by GeneNetwork) were taken from a list of all protein coding human genes (downloaded from the HUGO gene nomenclature committee website [68, 69]), and the overlap between these two samples recorded. This was repeated 1,000,000 times and the significance value was calculated by dividing the number of times the overlap between the two samples was greater than 1579 (the overlap we see) by the number of permutations (1,000,000).

## Electronic supplementary material

Additional file 1: **Genes with significant p-value (p ≤ 0.05) associated with hippocampus weight in BXD.** A table showing the human homologue gene symbols for all the mouse genes with a genome wide p-value ≤ 0.05 for hippocampus weight in BXD, showing their unadjusted p-value in human. (XLSX 11 KB)

Additional file 2: **Genes which are commonly coexpressed with** ***MGST3*** **as determined by GeneFriends.** A table showing the genes which are commonly coexpressed with *MGST3*, independent of tissue or treatment, as identified by GeneFriends. For each gene its Entrez gene ID, gene symbol and coexpression value with *MGST3* are shown. (XLSX 225 KB)

Additional file 3: **KEGG pathway annotations enriched in genes that are coexpressed with** ***MGST3*** **.** A table showing KEGG pathway annotations significantly enriched (calculated by DAVID) in genes that are significantly coexpressed with *MGST3* (calculated by GeneFriends). The table shows the category of the enrichment and the specific annotation, the number of submitted genes in the annotation/total number of submitted genes in the category, the fold enrichment and the FDR. (XLSX 10 KB)

Additional file 4: **Probes which correlate with all six of the correlating probes for** ***Mgst3*** **in the adult mouse hippocampus as determined by Pearson correlations in GeneNetwork.** A table showing the probes which correlate with all six of the *Mgst3* probes which correlate together. For each probe, the probe ID, Entrez gene ID of the mouse gene, Entrez gene ID of the homologous gene, the mouse gene symbol and the gene’s location is given. (XLSX 308 KB)

Additional file 5: **KEGG pathway annotations enriched in genes that are coexpressed with** ***Mgst3*** **in the adult mouse hippocampus.** A table showing KEGG pathway annotations significantly enriched (calculated by DAVID) in genes significantly coexpressed with *Mgst3* (calculated by Pearson correlation in GeneFriends). The table shows the category of the enrichment and the specific annotation, the number of submitted genes in the annotation/total number of submitted genes in the category, the fold enrichment and the FDR. (XLSX 10 KB)

Additional file 6: **Genes which commonly coexpress with** ***MGST3*** **as determined by GeneFriends and which coexpress with** ***Mgst3*** **in the adult mouse.** A table showing the homologous genes which commonly coexpress with *MGST3*, independent of tissue or treatment (as identified by GeneFriends) and those which are coexpressed with *Mgst3* in the adult mouse hippocampus, as determined by Pearson correlation in GeneNetwork. For each gene, its Human gene symbol, Human Entrez gene ID, Human chromosome, Human gene location, Mouse Gene Symbol, Mouse Entrez Gene ID, Mouse chromosome and Mouse gene location is shown. (XLSX 132 KB)

Additional file 7: **KEGG pathway annotations enriched in genes which are coexpressed with MGST3 (as determined by GeneFriends) and those which coexpress with** ***Mgst3*** **in the adult mouse.** A table showing the homologous genes which commonly coexpress with *MGST3*, independent of tissue or treatment (as identified by GeneFriends) and which coexpress with *Mgst3* in the adult mouse hippocampus, as determined by Pearson correlation in GeneNetwork. The table shows the category of the enrichment and the specific annotation, the number of submitted genes in the annotation/total number of submitted genes in the category, the fold enrichment and the FDR. (XLSX 10 KB)

## Abbreviations

BXD: A large set of recombinant inbred strains derived by crossing C57BL/6 J and DBA/2 J and inbreeding progeny for 20 or more generations.
CEU: Utah residents with Northern and Western European ancestry
DAVID: Database for annotation, visualization and integrated discovery
Df: Degree of freedom
ENIGMA: Enhancing neuro imaging genetics through meta-analysis
eQTL: Expression quantitative trait loci
FDR: False discovery rate
GO: Gene ontology
GWAS: Genome wide association study
HUGO: Human genome organisation
KEGG: Kyoto encyclopedia of genes and genomes
LD: Linkage disequilibrium
LRS: Likelihood ratio statistic
MGI: Mouse genome informatics
*MGST3*: Microsomal glutathione S-transferase 3 [Entrez 4259]
*Mgst3*: Microsomal glutathione S-transferase 3 [Entrez 66447]
MRI: Magnetic resonance imaging
mRNA: Messenger ribonucleic acid
QTL: Quantitative trait loci
SNP: Single nucleotide polymorphism
UCSC: University of California, Santa Cruz
UTR: Untranslated region
VEGAS: Versatile gene-based association study.
